# Perioperative coronary artery spasm after off-pump coronary artery bypass grafting in the non-manipulated coronary artery

**DOI:** 10.1186/s12872-022-02609-6

**Published:** 2022-04-12

**Authors:** Yunpeng Bai, Yiming Bai, Nan Jiang, Qingliang Chen, Zhigang Guo

**Affiliations:** 1 Department of Cardiac Surgery, Chest Hospital, Tianjin University, No. 261 Taierzhuang South Road, Jinnan District, Tianjin, 300222 China; 2 Department of Cardiac Surgery, Tianjin Chest Hospital, Tianjin Medical University, Tianjin, 300222 China; 3 Graduate School, Tianjin Medical University, Tianjin, 300070 China

**Keywords:** Coronary artery spasm, Coronary artery bypass grafting, Angiography, Calcium channel blocker

## Abstract

**Background:**

Perioperative coronary artery spasm (CAS) following coronary artery bypass grafting (CABG) is a severe or lethal condition that is rarely reported. In addition, rare cases with CAS following CABG in the non-manipulated coronary artery are angiographically documented in the perioperative period. We aimed to report our experiences on the diagnosis and treatment of a case with CAS following off-pump CABG in the non-manipulated coronary artery.

**Methods:**

A 57-year old male with coronary heart disease and unstable angina willing to undergo CABG was admitted to our department. CABG was recommended as he showed 90% stenosis in distal left anterior descending artery, 90% stenosis in intermediate branch, 90% stenosis in left circumflex coronary artery, as well as 50% stenosis in proximal right coronary artery (RCA).

**Results:**

After CABG, the patient showed Adams–Stokes syndrome and ST-segment elevation. Then CPR was conducted and coronary angiography indicated perioperative CAS in the non-manipulated posterior descending artery. For the treatment, the patient received nitroglycerin injection into the coronary artery by catheter and pumping of diltiazem. Finally, the patient was discharged on day 7 after surgery. A comprehensive literature search was conducted to summarize the studies focused on the diagnosis and treatment of such condition, which indicated that all of the CAS cases occurred in the manipulated vessels, except one study showing CAS in the untouched native coronary artery which was similar with our case.

**Conclusions:**

Perioperative CAS in the non-manipulated coronary artery following CABG is a severe or lethal condition that is rarely reported, which deserves close attention by the clinicians in clinical practice.

**Supplementary Information:**

The online version contains supplementary material available at 10.1186/s12872-022-02609-6.

## Background

Coronary artery spasm (CAS) is defined as a temporary tightening or constriction of the muscles in the arterial walls, which can decrease or completely block the blood flow to heart [1, 2]. Patients with CAS may present angina or chest pain, or even myocardial infarction.

Coronary artery bypass grafting (CABG) is a surgical procedure where atheromatous blockages in a patient’s coronary arteries are bypassed with harvested arteries or veins [3]. According to a recent survey, approximately 400,000 CABG surgeries are performed worldwide annually. Among these patients, CAS is a known complication following CABG, with an incidence of 1–8% [4, 5].

To our best knowledge, very rare patients with CAS following CABG in the non-manipulated coronary artery are angiographically documented in the perioperative period [5, 6]. Additionally, there is still a lack of treatment guideline as the pathogenesis of CAS following CABG is still not well defined. In this study, we reported our experiences on the diagnosis and treatment of CAS following off-pump CABG in the non-manipulated coronary artery, and a comprehensive literature search was conducted to summarize the diagnosis and treatment experiences on the published cases.

## Patient and method

### Patient

The patient was a 57-year old gentleman who was admitted to our department due to coronary heart disease and unstable angina. He had a smoking history for at least 30 years, with a frequency of 20 cigarettes per day.

### Diagnosis and treatment

Based on the patient’s conditions, coronary angiography was conducted by experienced clinicians in our department. Coronary angiography was conducted to investigate the vascular conditions. According to the patient’s conditions on admission, the patient was suggested to undergo off-pump CABG. Therefore, contraindications for surgical procedures such as off-pump CABG were carefully checked. The patient received oral administration of isosorbide mononitrate tablet, metoprolol tartrate and atorvastatin calcium tablets, as well as subcutaneous injection of low molecular heparin.

### Surgical technique

The whole procedures were conducted under general anesthesia. The sternum was split through the median incision. The great saphenous vein served as the vein graft. Upon exposure of the heart, off-pump CABG was performed to explore the in situ vessels of coronary artery. The Stable-PV1-1 device was utilized for the fixation of anterior descending branch and intermediate branch, followed by revascularization. Upon revascularization, the vascular flow in the internal mammary artery graft was 25.4 mL/min, and the pulsatility index was 3.1. The vascular flow in the intermediate branch vein graft was 28.2 mL/min. The pulsatility index was 2.9.

### Follow-up

The patient was followed up for 12 months. Regular follow-up was suggested to the patient. Additionally, the patient was informed to visit our department in cases of any discomforts. The complications and the cerebrovascular events were recorded after face-to-face communication.

## Results

### Patient conditions

**Fig. 1:**
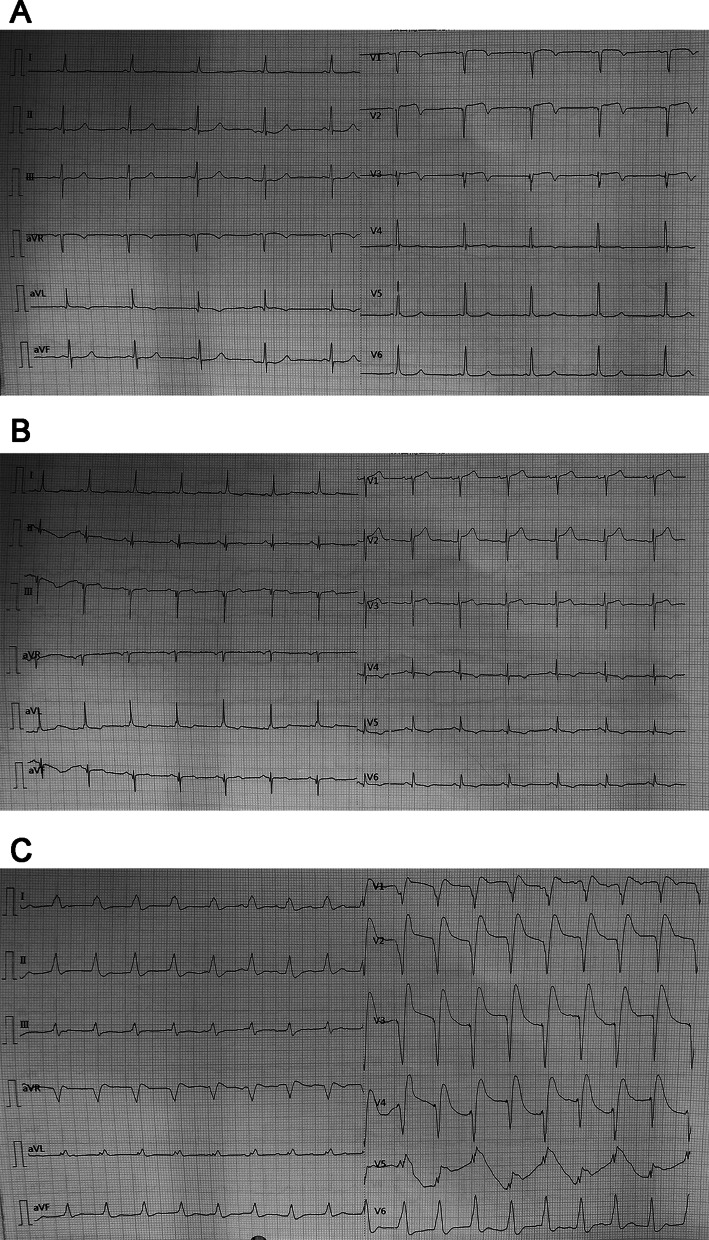
ECG findings of the patient. **A** Preoperative ECG. **B** ECG performed after removal of respirator at postoperative 17 h. **C** ECG performed at postoperative 23 h when the patient showed Adams–Stokes syndrome

Coronary angiography revealed distal stenosis (90%) in the left anterior descending artery (LAD), intermediate branch stenosis (90%), left circumflex coronary artery stenosis (90%), as well as proximal stenosis in the right coronary artery (RCA, 50%) (Additional files 1–4). Then off-pump CABG was conducted with LAD revascularization using left internal mammary artery and intermediate branch revascularization using great saphenous vein. The left circumflex was tenuous and the vascular conditions were poor, and then no bypass was given. In addition, no bypass was given to the right coronary artery as the lesions were slight. About 10 h after operation, the patient's vital signs were stable and then the ventilator was removed. About 23 h after off-pump CABG, the patient showed Adams-Stokes syndrome, and the blood pressure showed decrease. In addition, pathological Q Waves (II, III, aVF) appeared, together with ST-segment elevation in the ECG (Fig. 1). Moreover, the concentration of creatine kinase-MB (CK-MB) and high-sensitivity cardiac troponin T (hs-cTnT) showed significant increase, indicating a possibility of acute myocardial infarction (Table 1). Furthermore, the heart rate showed decline. On this basis, cardiopulmonary resuscitation was given immediately, followed by mechanical ventilation and blood pressure management using vasoactive drugs. Finally, intra-aortic balloon pump (IABP) was implanted.

**Table 1:** Concentration of hs-cTnT and CK-MB at four time points

Marker	Post-operative 4 h	Post-operative 18 h	Post-operative 23 h	Post-operative 48 h	Post-operative 7d
hs-cTnT, ng/ml	0.086	9.870	8.38	4.96	0.82
CK-MB, U/L	19	159	70	64	18

### Observation of CAS

Subsequently, the patient was transferred to the Hybrid Operation Room (Hybrid-OR), and received post-operative coronary angiography showed no obstruction in the grafts involved in the surgery (Additional file 5 and 6). In addition, there was occlusion in the orifice of the posterior descending branch of the RCA which is the non-manipulated coronary artery (Additional file 7). The patient was finally diagnosed with CAS following CABG the non-manipulated coronary artery.

### Treatment and follow-up

**Fig. 2:**
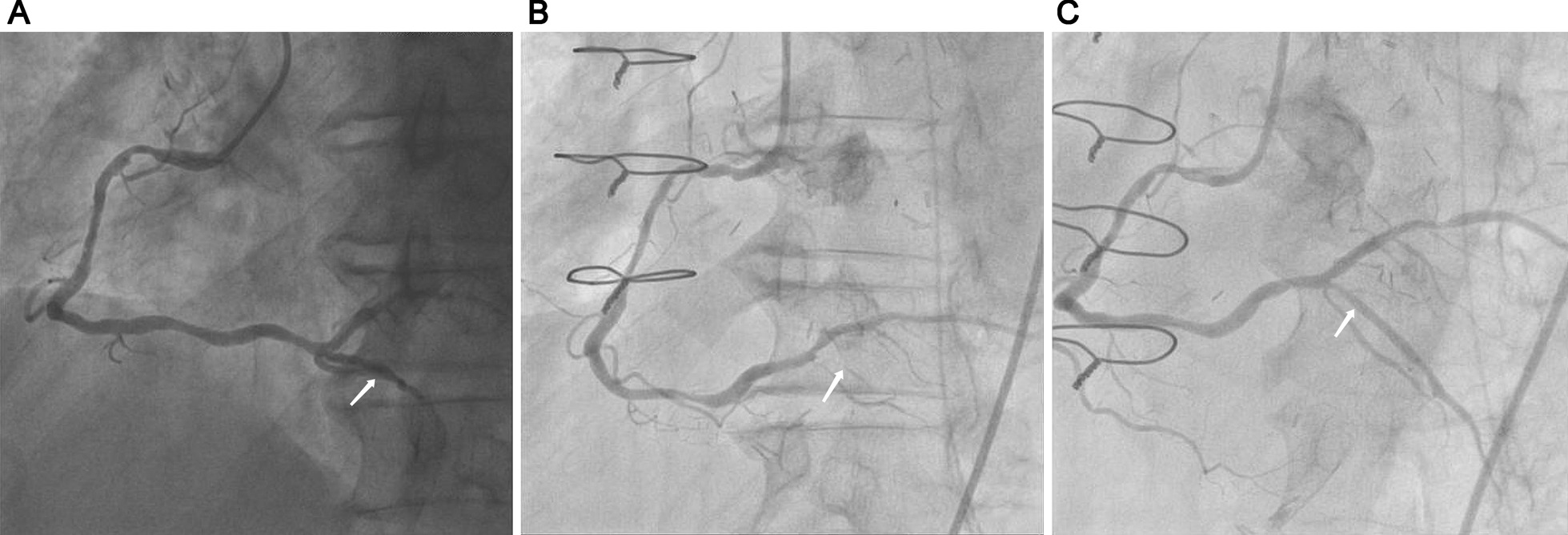
Preoperative and postoperative coronary angiography. **A** Preoperative angiography indicated 50% stenosis in right main coronary artery. **B** Angiographic findings of right coronary artery after onset of Adams-Stokes syndrome. **C** After administration of nitroglycerin into the spasm site, the posterior descending branch was visualized. White arrow indicated the spasm in the orifice of the posterior descending branch in the non-manipulated vessel

For the treatment of spasm, perioperative infusion of nitroglycerin was given through the angiographic catheter. Then the spasm showed complete attenuation as revealed by the angiography that was conducted about 5 min after the nitroglycerin infusion through catheter (Fig. 2 and Additional file 8). The clinical signs were stable and the heart function showed gradual recovery. The ventilator and IABP were removed on postoperative day 3 and 4, respectively. The level of CK-MB was recovered to the normal range, and the hs-cTnT showed significant decrease on postoperative day 7. The patient received intravenous infusion of drugs early after operation, and gradually transition to oral administration of diltiazem after surgery (30 mg, t.i.d.). The medications used in the follow-up included aspirin, clopidogrel, atorvastatin calcium, metoprolol tartrate and diltiazem. ACS remained to be associated with higher BARC 3 or 5 bleeding risk, and then the patient received Pantoprazole after operation [7]. He was followed up for 12 months, and showed a normal status with no cerebrovascular events. No revascularization was required during the 12-month follow-up, and no angina was reported by himself.

## Discussion

CAS in the early postoperative phase after CABG has been considered an unrecognized cause of sudden and severe cardiopulmonary collapse [8]. It usually occurs within 24 h after surgery, and is responsible for about one third of the instances of early postoperative circulatory collapse [6, 9]. To our best knowledge, several risk factors have been identified in cases experiencing postoperative CAS, among which the preoperative cardiac angina is considered an important identification factor [10, 11]. Cases presenting initial signs of CAS usually show acute hypertension, and some patients (8%) are likely to present transient 2-mm or greater ST segment elevation on the ECG, which has been acknowledged as the most important diagnostic standards for CAS [12]. In this study, we analyzed the published articles regarding perioperative CAS, and the methods of prevention and diagnosis. Our study contributed to the diagnosis and treatment of CAS especially in those underwent CABG.

### Risk factors inducing CAS

Smoking, age and high-sensitivity C-reactive protein (CRP) have been reported as significant risk factors for CAS. The aged cases are more likely to develop CAS than the younger counterparts [13]. In addition, Brunner et al. showed that smoking would trigger vascular injury, decline of coronary arterial flow and partial pressure of oxygen in myocardium, which were considered to be participated in the onset of CAS [14]. In cases with low hs-CRP, diabetes mellitus has been shown to contribute to CAS development in men rather than women. Moreover, high cholesterol and high/low density lipoprotein may be directly associated with the contraction of the coronary artery. For instance, there was a positive correlation between coronary arterial contraction and total cholesterol and low density lipoprotein during the CAS [15]. High concentration of low density lipoprotein would affect the prognosis of CAS patients after administration of calcium channel blockers (CCBs) [16]. Therefore, dyslipidemia was also a risk factor for the pathogenesis of CAS. Furthermore, the aged population are more apt to develop CAS which has been found to be the reduced vasodilatation mediated by nitric oxide, increase of ROS, vascular endothelial dysfunction, decline in the fibrinolysis and the concurrent atherosclerosis [17]. Furthermore, a large number of male patients would have to undergo PCI after CABG [18]. In this study, the patient was an aged male with increase of triglyceride, total cholesterol and low density lipoprotein compared with the normal range, which may explain to the onset of CAS.

### Reasons inducing arterial spasm following CABG

To date, the etiology of CAS following CABG is still not well defined. Some postoperative factors are reported to affect the CAS, including vascular trauma, platelet activation, high local potassium [1, 19]. The occurrence of CAS showed no specific sites, such as a site of mild or severe stenosis, or any segment of the coronary arteries with no structural anomaly. Interestingly, studies have stated that the lesions at spasm sites have less plaque, no calcification, more diffuse intimal thickening, less lipid and necrotic core, thicker baseline medial width, more prevalent negative remodeling, less thin cap fibrous atheroma, and very small baseline luminal area. Therefore, CAS is considered to be associated with vasoconstrictor stimuli, and smooth muscle cell hyper-reactivity, as well as mechanical stimuli. It has been well acknowledged that the CAS is closely related to several signaling pathways, especially those involving concentration regulation of the intracellular calcium. As the CABG involving a rather complicated procedures and potential injuries and/or manipulation on the blood vessels and organs, there might be changes in the calcium ion concentration, and endothelial function that are considered to be crucial for the pathogenesis of vasospasm. In addition, manipulation on the vessels could indeed induce a high incidence of vascular spasm. As described in the Tarhan et al. study [20], donor vessel should be manipulated carefully to minimize surgical trauma during the harvest. However, these may raise the possibility of CAS inevitably. According to the literature search in our study, all of the CAS cases occurred in the manipulated vessels, except one study showing CAS in the untouched native coronary artery [21]. In our case, the spasm following CABG occurred in the non-manipulated RCA, which was the second case with CAS in the untouched native coronary artery.

### Literature search on CAS after CABG

To date, less than 20 CAS cases from 10 articles [4, 8, 21–28] have been reported after CABG based on a complete literature review in the PubMed, Medline, Embase databases, using the following key words: coronary artery spasm, Prinzmetal angina, coronary vascular spasm, coronary vasospasm, or coronary artery vasospasm. Only the articles with full text published in English languages were selected (Table 2). In a previous study in 1984, Skarvan et al. reported 10 cases with CAS following CABG [29]. However, the clinical characteristics of each patient was not illustrated in a detailed manner. Therefore, the study was not included in the literature review. Based on the literature review, we found that most of the cases were aged 40 or more, with a male predominance than women. For the preoperative conditions of the CAS patients, most showed angina (or chest pain) and stenosis in coronary artery with up to 50% or more. These patients were suggested to undergo CABG and symptomatic treatment. The majority of cases showed ST-segment elevation as revealed by ECG, together with hypertension. Most of the vasospasm were not detected in a real-time manner, but was diagnosed based on the relieve of spasm-related conditions after anti-spastic agents. In our case, the CAS was detected upon onset based on angiography. According to the literature search, there is no favor for the vessels affected by spasm. It seemed that most of the cases with CAS showed vasospasm in the manipulated vessels. Only one study reported a case of CAS in the non-manipulated vessel. In our case, the spasm was approved to be localized in the posterior descending branch of the non-manipulated coronary artery after angiography. The preoperative angiography indicated eccentric stenosis in posterior descending branch. This is an extremely rare condition among the detected CAS following off-pump CABG. To explain such phenomenon, we speculated the following aspects: (i) the patient suffered from coronary atherosclerosis and cerebral infarction; (ii) the surgical procedures would trigger the nerve-body fluid disorder and internal environment disorder; (iii) the patient was a smoker and dysplipidemia; (iv) eccentric stenosis; and (v) the administration of the anesthetic agent and hypoxia stimuli would trigger the excitation of the sympathetic nerves, which then resulted in the vascular smooth muscle cell contraction in the coronary artery, as well as elevation of calcium ion and the vasoconstrictor substance [30].

**Table 2:** Clinical characteristics of CAS after CABG

Patient	Year of publication	Symptoms and preoperative findings	Evidence of postoperative CAS	Treatment	Patient outcome
1	1981 [8]	Exertional angina, 90% obstruction in the left main coronary artery	Inferior ST-segment elevation, hypotension	Intravenous NTG, IABP, nifedipine	Died
2	1981 [8]	Exertional and variant angina, 80% obstruction in the left anterior descending artery	Inferior ST-segment elevation, hypotension, sinus bradycardia	Intravenous NTG, IABP	Died
3	1981 [8]	Variant angina, 95% obstruction of LADA	Inferior ST-segment elevation, hypotension	Intravenous NTG, nifedipine	Died
4	1981 [8]	Rest and exertional angina, 90% obstruction in left main coronary artery	Inferior ST-segment elevation, hypotension, ventricular tachycardia	NTG, nifedipine	Recovery, but not reporting the discharge conditions
5	1981 [8]	Exertional angina, 99% obstruction of LADA, 90% obstruction of LCx	Inferior ST-segment elevation, hypotension, and atrioventricular block	NTG, nifedipine, IABP	Recovery, but not reporting the discharge conditions
6	1981 [8]	Rest and exertional angina, 90% obstruction of LADA, 70% obstruction of diagonal artery	Inferior ST-segment elevation, hypotension, and atrioventricular block	NTG, nifedipine, phentolamine	Recovery, but not reporting the discharge conditions
7	2007 [23]	Recurrent angina, underwent PTCA of the LCx and RCA; critical lesions of LCx and RCA	ST-segment elevation over leads II, III and aVF by ECG	Verapamil and NTG into the vein grafts by intracoronary injection, followed by i.v. infusion of nitroprusside and NTG	Discharged on day 10 uneventfully after surgery
8	2007 [23]	Exertional angina, left main coronary artery stenosis	ECG showed ST-segment elevation on lead III, short-run ventricular tachycardia	IABP and infusion of inotropic agent	Discharged on day 9 uneventfully with good clinical conditions
9	2007 [23]	Chest tightness, underwent PTCA with RCA stenting; total LAD occlusion and in-stent restenosis of RCA	ECG showed Q waves over leads II, III, and aVF, as well as inverted T wave over V4-V6	ECMO, intracoronary and systemic administration of NTG and nitroprusside; bolus injection of epinephrine and IABP insertion	Discharged with no complications on day 20 after surgery
10	2003 [26]	underwent PTCA with stenting of Cx and RCA; pre-stent sub-occlusive lesion of the RCA	ST segment elevation in lead D3, reduction of R wave in lead aVF, D3 and ST segment depression in lead V3-6, D1 and AVL; a new akynetic area in inferior left ventricular wall	Insertion of an IABP and intravenous NTG infusion	Discharged on 12 after surgery in good clinical conditions; survived in the 3-year follow-up
11	1999 [22]	Exertional angina, hypertension and previous myocardial infarction; angiogram revealed a mildly impaired ventricular contractility, a 90% stenosis on the LAD involving the first diagonal branch, and a 90% proximal stenosis in the RCA	Antero-lateral myocardial ischemia, elevation of blood pressure (33/30 mmHg) associated with ST-segment depression	Diltiazem, glyceryl trinitrate; intracoronary infusion of nitrates and verapamil	Discharged on day 7 after surgery
12	2013 [24]	Sudden bradycardia, hypertension; stenosis in the proximal anterior descending branch of LCA; 90% stenosis in the first and second diagonal branch, 90% stenosis of obtuse marginal branch and high lateral artery	ST segment elevation	Diltiaze, NTG, NCR, IABP	Discharged with no complications on day 74after surgery
13	2007 [25]	Recurrent angina; 60% in-stent stenosis with extension into the left main and ostial LCx	Minimal spasm in the radial artery graft, worsening of the native LAD lesion, and diffuse spasm of the native RCA	IABP, dobutamine and milrinone	Discharged on day 8 after aftery
14	2005 [27]	Frequent angina; 90% stenosis in left main trunk and 75% stenosis of posterolateral brunch of RCA	CAS was not observed, but was speculated based on ECG and hemodynamic deterioration. ST segment depression in precordial leads on ECG, ST-segment elevation in II, III and aVF leads	Diltiazem, vasodilator agents such as nicorandil, verapamil, papaverine and NTG; IABP	Not reported
15	2010 [4]	Unstable angina; 80% obstruction of proximal left AIA	Extensive anterior ischemia after ECG, ST segment elevation	Intracoronary vasodilators	Discharged on 13 after surgery
16	2010 [21]	Chronic stable angina pectoris and hypertension; 90% stenosis on distal left main coronary artery	ST segment depression, hypertension,	Intracoronary infusion of NTG, oral medication of nicorandil	Discharged on 15 after surgery
17	1990 [28]	Angina and chest pain at rest, nocturnal pain; multiple stenosis in RCA, Cx and LAD	A supranodal rhythm and an incomplete right bundle branch block, with ST elevation in V3 and V4	GTN, intravenous nifedipine, dobutamine	Free of angina in the 1-year follow-up

*LAD* left anterior descending artery; *NTG* nitroglycerin; *PTCA* percutaneous coronary angioplasty; *LCx* left circumflex; *RCA* right coronary artery; *aVF* augmented voltage foot; *IABP* intraaortic balloon pump; coronary angiography; *LCA* left coronary artery; *NCR* nicorandil; *ECG* electrocardiogram; *AIA* anterior interventricular artery; *GTN* glyceroltrinitrate

Indeed, there are still disputes on the necessity of bypass grafting in the vessels with lesions that are not severe. For this case, the lesions in the RCA were not severe, and the bypass grafting was not conducted, however, there might be possibility of perioperative CAS. In cases of bypass grafting in RCA with non-severe lesions, there might be a high possibility of competitive blood flow in the involved vessels and the bypass vessels, which may lead to severe stenosis in the RCA. In future, more studies are required to illustrate the necessity of bypass grafting for the patients presenting non-severe lesions in the RCA.

### Suggested treatment options for CAS

The goal of CAS treatment is to control chest pain and prevent a heart attack. Nowadays, there are no uniformed treatment options for treating CAS after CABG. The management of CAS in clinical practice is highly relied on the nitrates, CCBs and statin medications [5]. Nitroglycerin could relax the smooth muscles and the subsequent relief of the spasmodic pain. In hypertensive subjects received nitroglycerin, there was decrease in blood pressure as it could serve as a vasodilator to open blood vessels to improve the blood flow. To date, three CCB agents, including nifedipine, verapamil, and diltiazem [31], are important adjuncts to treatment of spasm, management of blood pressure and arrhythmia. CCBs are beneficial to myocardial oxygen supply in CAS patients, and are also useful in the prevention of classic exertional angina caused by fixed obstruction. For instance, diltiazem was used with a 30 mg dose, because in some patients with relatively low blood pressure, side effects started at a lower dose of 30 mg per day [32]. In a comparative study focused on the efficiency of diltiazem along versus its combination with nitrate, the combination of diltiazem and nitrate was not superior to diltiazem in reducing mortality and cardiovascular events in the 5-year follow-up among the CAS patients despite the fact that the combination contributed to the improvement of endothelial function and relief of CAS [33]. In this study, the spasm showed complete attenuation as revealed by the angiography that was conducted about 5 min after the nitroglycerin by transcatheter infusion. The patient received intravenous infusion of drugs early after operation, and gradually transition to oral administration of diltiazem (30 mg, t.i.d.) after surgery.

There are still some limitations in this study. Despite the fact that CAS would lead to severe or even lethal conditions in patients, it is still a challenge to determine the incidence of CAS serving as a cause for postoperative death as most cases of this phenomenon in the studies were survivors. However, it may function as an important contributor to early postoperative mortality than the previous recognition [29]. Only three cases were died after CAS following the CABG, while the others were survived. As these patients may present other severe complications, it is hard to illustrate the exact causes for the CAS.

## Conclusion

Perioperative CAS following CABG in the non-manipulated coronary artery has been rarely angiographically documented. In this study, we reported a case of CAS following off-pump CABG in the non-manipulated coronary artery. In addition, based on the literature review, we summarized the symptoms and the imaging findings of these patients, which may contribute to the diagnosis of such lethal conditions in clinical practice. Moreover, we summarized the treatment regimens and the prognosis of these patients. Our study would provide sufficient information for the clinical management of CAS following CABG.

## Supplementary Information

**Additional file 1**: Preoperative angiography for the right coronary artery. The video was displayed in a 35 projection degree for LAO and 1 projection degree for CRA.

**Additional file 2**: Preoperative angiography for the right coronary artery. The video was displayed in a 7 projection degree for RAO and 25 degree for CRA.

**Additional file 3**: Preoperative angiography for the left coronary artery. The video was displayed in a 31 projection degree for RAO and 31 degree for CRA.

**Additional file 4**: Preoperative angiography for the left coronary artery. The video was displayed in a 28 projection degree for LAO and 13 degree for CRA.

**Additional file 5**: Postoperative angiography for the left internal mammary artery and LAD.

**Additional file 6**: Postoperative angiography for the great saphenous vein, and intermediate branch.

**Additional file 7**: Postoperative angiography indicated the presence of CAS in the orifice of posterior descending branch of the RCA.

**Additional file 8**: The CAS showed improvement after nitroglycerin injection into the right coronary artery by catheter.

## Data Availability

All data generated or analysed during this study are included in this published article.

## Abbreviations

CAS: Coronary artery spasm
CABG: Coronary artery bypass grafting
IABP: Intra-aortic balloon pump
Hybrid-OR: Hybrid operation room
CCBs: Calcium channel blockers
